# Self‐organization of active plume lattice in bacterial bioconvection

**DOI:** 10.1002/qub2.80

**Published:** 2024-12-18

**Authors:** Siyu Liu, Qihui Hou, Daniel B. Kearns, Yilin Wu

**Affiliations:** ^1^ Department of Physics and Shenzhen Research Institute The Chinese University of Hong Kong Hong Kong China; ^2^ Department of Biology Indiana University Bloomington Indiana USA

**Keywords:** bioconvection, chemotaxis, microswimmers, pattern formation, self‐organization

## Abstract

Self‐organized pattern formation is common in biological systems. Microbial populations can generate spatiotemporal patterns through various mechanisms, such as chemotaxis, quorum sensing, and mechanical interactions. When their motile behavior is coupled to a gravitational potential field, swimming microorganisms display a phenomenon known as bioconvection, which is characterized by the pattern formation of active cellular plumes that enhance material mixing in the fluid. While bioconvection patterns have been characterized in various organisms, including eukaryotic and bacterial microswimmers, the dynamics of bioconvection pattern formation in bacteria is less explored. Here, we study this phenomenon using suspensions of a chemotactic bacterium *Bacillus subtilis* confined in closed three‐dimensional (3D) fluid chambers. We discovered an active plume lattice pattern that displays hexagonal order and emerges via a self‐organization process. By flow field measurement, we revealed a toroidal flow structure associated with individual plumes. We also uncovered a power‐law scaling relation between the lattice pattern’s wavelength and the dimensionless Rayleigh number that characterizes the ratio of buoyancy‐driven convection to diffusion. Taken together, this study highlights that coupling between chemotaxis and external potential fields can promote the self‐assembly of regular spatial structures in bacterial populations. The findings are also relevant to material transport in surface water environments populated by swimming microorganisms.

## 1 INTRODUCTION

Self‐organized pattern formation is a hallmark of biological systems [1]. Microbial populations can generate large‐scale regular spatial patterns via biological or physical mechanisms such as chemotaxis [2], quorum sensing [3, 4, 5], and mechanical interactions [6, 7, 8]. When their motile behavior is coupled to an external gravitational potential field, various microorganisms including algae, protozoa, and bacteria display a phenomenon known as bioconvection [9, 10]. Bioconvection normally occurs in shallow layers of fluids populated by microorganisms through coupling of taxis motion, buoyancy, and fluid flows [9]: Due to the presence of oxygen or light intensity gradients in the fluid layer or simply due to the gravitactic capability, microbial cells tend to swim upward and aggregate near the top of the fluid layer. The cell aggregates would then drift downward via an overturning instability because the mass density of cells is higher than the ambient fluid, producing vertically circulating flows and leading to domains of higher cell density. The high‐density domains are reminiscent of fluid plumes seen in thermal Rayleigh–Bénard convection of passive fluids [11] and they are referred to as active cellular plumes in this study. Bioconvection enhances fluid mixing, and it is believed to play a role in material transport or resource distribution during microbial processes in surface water environments such as algae blooms [12, 13].

Although bioconvection has been found in various microorganisms, the dynamics of bioconvection pattern formation in bacteria is relatively less explored [14, 15]. Here, we study this phenomenon using suspensions of a chemotactic bacterium *Bacillus subtilis* (*B. subtilis*) confined in closed three‐dimensional (3D) fluid chambers in the presence of oxygen gradients. We discovered an active plume lattice pattern that displays a hexagonal order and emerges via a self‐organization process with migration, merging, and birth of plumes. Our flow field measurement revealed a toroidal flow structure associated with individual plumes. We further studied how physical parameters (cell number density and fluid thickness) control the plume lattice pattern. Our findings highlight that coupling between taxis behavior and external potential fields can promote the self‐assembly of regular spatial structures in bacterial populations. It may shed light on the control of pattern formation and material transport in active matter fluids [16] consisting of natural or synthetic microswimmers [17].

## 2 RESULTS

### 2.1 Ordered active plume lattice emerges in 3D confined *B. subtilis* suspensions

**FIGURE 1:**
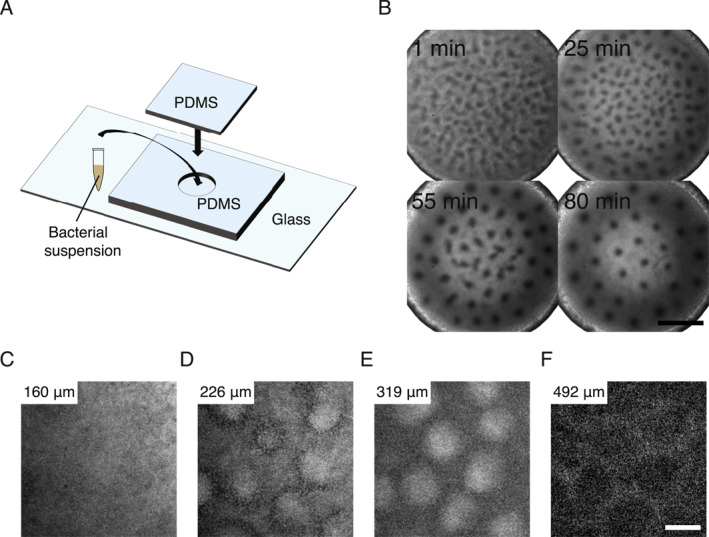
Emergence of bacterial plume lattice pattern in *B. subtilis* dense suspensions. (A) Schematic diagram of the experimental setup for the formation of bacterial plume lattice pattern. (B) Representative phase‐contrast microscopy image sequence showing the spontaneous formation of a regular lattice of bacterial plumes in a 3D microfluidic chamber. The plumes appear as darker regions against a lighter background due to higher cell densities (Figure S1). *T* = 0 min corresponds to the starting point of observation immediately after filling the bacterial suspension into the microfluidic chamber. Scale bar, 1 mm. (C–F) Confocal microscopy images showing the spatial distribution of cell number density at different heights within a chamber of thickness ∼500 μm. (C) (160 μm, height measured from the chamber floor): The cell density distribution in the focal plane was uniform; (D) (226 μm): Higher‐density regions began to appear and they were surrounded by lower‐density regions; (E) (319 μm): Cell density inhomogeneity became more prominent; (F) (492 μm, near the top surface): Cell density inhomogeneity was less prominent, presumably due to the no‐slip boundary condition that prevents the replenishment of cells from surroundings to the core of active plumes. The higher cell‐density regions form distinct columnar structures characteristic of bacterial active plumes. Each image from panel (C–F) has a different contrast or dynamic range, as the fluorescence emission near the upper surface may be partially absorbed by the bulk of the bacterial suspension. Panels (C–F) share the same scale bar (100 μm).

To investigate the dynamics of bioconvection pattern formation in bacteria, we chose to work with the model bacterium *B. subtilis*, which exhibits robust aerotactic behavior (a special form of chemotaxis) [18] and bioconvection phenomenon [14, 15]. We used *B. subtilis* *sin* *I* mutants defective in matrix production and biofilm formation to ensure that the cells have uniform motility (Methods) [19, 20]. The *B. subtilis* suspension (1.2 × 10^10^ cells/mL) was transferred into a closed polydimethylsiloxane (PDMS) microfluidic chamber (thickness ~500 μm, diameter ∼4 mm; Figure 1A); cell activities consumed oxygen in the fluid chamber and generated an oxygen gradient pointing upward vertically. Upon being transferred into the chamber, an initially homogeneous suspension developed a disordered distribution of domains that appeared as darker spots under phase‐contrast microscopy (Figure 1B, Video S1). Subsequently, the system underwent a self‐organization process, eventually forming a highly ordered hexagonal lattice over the course of ∼1 h (Figure 1B, Video S1).

Fluorescence imaging showed that these darker domains under phase‐contrast microscopy were of a higher cell density than nearby regions (Figure S1). As revealed by confocal microscopy, each of these domains consisted of a high cell‐density column spanning ∼250–300 μm vertically (Figure 1C–F), which is characteristic of active cellular plumes in bioconvection patterns [14] (Figure S2). The lattice pattern of high cell‐density domains was abolished when a nonchemotactic mutant of *B. subtilis* (*cheB* mutant; Methods) was used in the experiment or when the oxygen gradient direction was inverted to be aligned with the direction of gravitational force (by inverting the fluid chamber), demonstrating an essential role of chemotactic behavior in the lattice pattern formation. These results showed that the observed pattern of high‐density domains is indeed a form of bioconvection; hence, we referred to the pattern as active plume lattice.

### 2.2 Developmental process of the hexagonal active plume lattice

**FIGURE 2:**
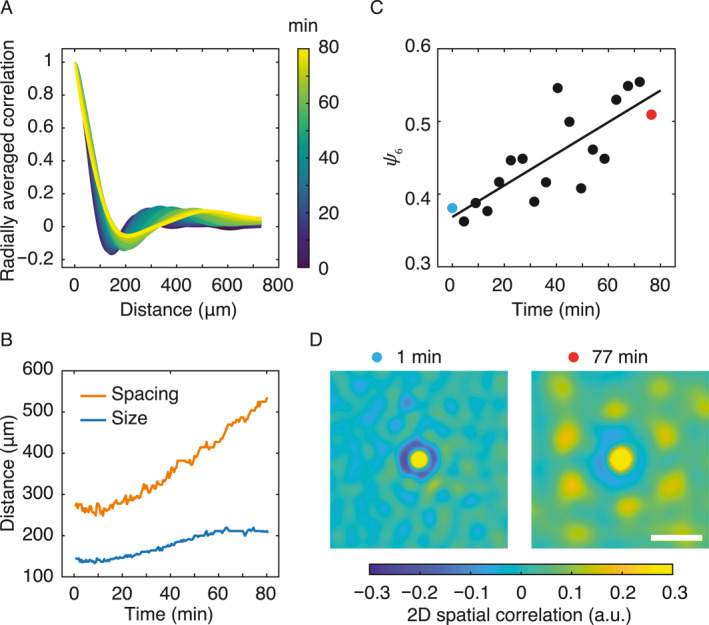
Spatiotemporal dynamics of bacterial plume lattice pattern development. (A) Temporal dynamics of radially averaged spatial correlation function of the bacterial plume pattern associated with Figure 1B. Color map indicates the time (unit: min). (B) Temporal evolution of the plume size (blue) and spacing between plumes (red). (C) Temporal evolution of hexagonal order parameter *ψ*_6_ of the bacterial plume pattern. Solid line is a least‐squares linear fit with a slope of ∼0.0022 (*R* ^2^ = 0.65). (D) 2D spatial correlation of the bacterial plume pattern associated with blue data point (left) and red point (right) in panel (C). Color bar is in arbitrary units (a.u.). Scale bar, 500 μm.

The developmental process of the observed active plume lattice pattern involves migration, merging, and birth of plumes. We computed the radially averaged spatial correlation function of plumes and plotted it over time (Figure 2A); the radially averaged spatial correlation function generally exhibits peaks and valleys, with the first local minimum representing the average size of the plumes and the first local maximum representing the average inter‐plume distance or the wavelength of the plume pattern (Methods). Analysis of the radially averaged spatial correlation function showed that the plumes initiated with an average diameter of ∼130 μm in an irregular configuration. Over the course of ∼80 min, while plumes migrated randomly and coalesced into larger ones, the plume diameter and inter‐plume spacing continuously increased to ∼200 μm and ∼500 μm, respectively (Figure 2B). Meanwhile, the plume arrangement became more and more regular, with the hexagonal order parameter, *ψ*_6_, increasing by ∼30% (Figure 2C; Methods). The hexagonal order of the plume arrangement was also demonstrated by the six‐fold symmetry in the 2D spatial correlations of the pattern’s phase‐contrast image (Figure 2D; Methods). We note that the bioconvection pattern previously reported in *B. subtilis* [14, 15] was less regular, with the plumes arranged in curtain‐like chains, which is presumably due to the presence of evaporation induced flows (also see Discussion).

### 2.3 Individual active plume exhibits a toroidal convection flow pattern

**FIGURE 3:**
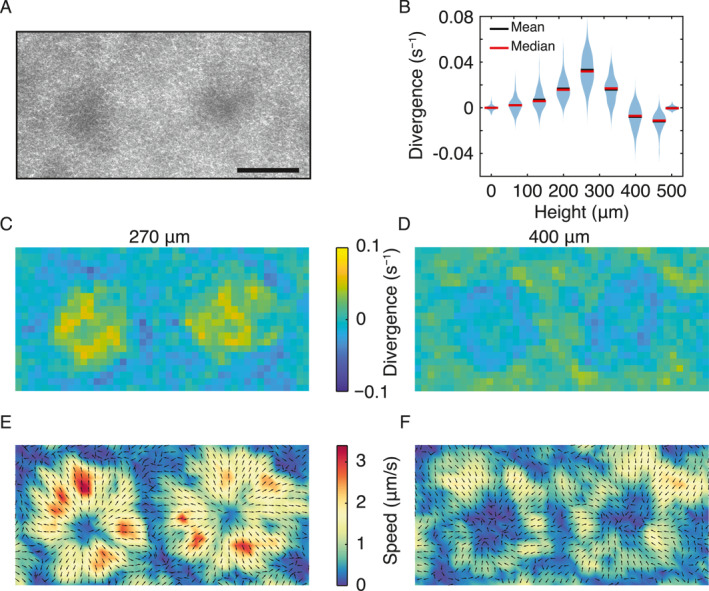
Fluid flow patterns within and surrounding bacterial plumes. (A) Phase contrast image of a representative pair of plumes selected for measuring the fluid flows. Darker region corresponds to the center of the two plumes. Scale bar, 200 μm. (B) Violin plot depicting the probability distribution of the pixelwise in‐plane divergence within plumes as a function of height above the bottom of the chamber. Black and red lines represent the mean and median divergence at each height, respectively. (C, D) Spatial distribution of in‐plane divergence at height of 270 μm (panel C) and 400 μm (panel D) above the bottom of the chamber. Colormap denotes magnitude of divergence. (E, F) Time‐averaged in‐plane flow velocity vector fields at heights of 270 μm (panel E) and 400 μm (panel F). Arrows and colormap represent flow velocity direction and magnitude, respectively.

To further characterize the structure of the active plumes in the lattice pattern, we measured the flow field associated with individual plumes (Figure 3A). Two‐dimensional (2D) flow fields were obtained at various vertical positions throughout the chamber by performing particle image velocimetry (PIV) with fluorescence images of tracer particles seeded in the bacterial suspension (Methods). The obtained flow fields allowed us to calculate the in‐plane divergence distribution. As shown in Figure 3B for flow fields within the plumes, the average in‐plane divergence first increased with vertical position, from near zero at the bottom of the chamber to a maximum of 0.03 s^−1^ at a height of ∼270 μm (i.e., near the mid‐plane of the fluid chamber), indicating an outward radial flow; above the height of ∼270 μm, the average divergence decreased with vertical position and became negative at heights ranging from ∼400 μm to ∼500 μm, indicating an inward flow toward the plumes at the upper quadrant of the plumes. It is worth noting that the mean divergence is zero at the bottom and uppermost surfaces due to the no‐slip boundary condition. This boundary condition plays a crucial role in determining the vertical velocity profile within the chamber.

To better understand the flow pattern, we examined the divergence distribution at two specific vertical positions where the average flow field divergence within the plumes had opposite signs, that is, ∼270 μm and ∼400 μm above the chamber bottom. At the height of ∼270 μm, where the flow divergence is positive within the plumes, the plumes were surrounded by a negative‐divergence valley (Figure 3C); accordingly, the fluid flows were radially moving outward from the plume centers and these outward flows converged in the middle of two neighboring plumes (Figure 3E). Conversely, at the height of ∼400 μm, where the flow divergence is negative within the plumes, the plumes were surrounded by a positive‐divergence ridge (Figure 3D); meanwhile, the fluid flows were radially moving inward toward the plume centers and these inward flows originated from the middle of two neighboring plumes (Figure 3F). This stratified flow structure, with the plumes acting as flow sources in the lower part and sinks in the upper part of the chamber, reveals a toroidal convection pattern associated with individual plumes.

We also analyzed the vertical flow speed (*v*_*z*_) across the chamber height *H*. Using the incompressibility condition ∇⋅**v** = 0, we obtained *v*_*z*_(*h*) by integrating the in‐plane divergence (*D*_in_) of fluid flow field as $v_{z}\left(H\right)=-\int_{0}^{H}D_{\text{in}}\left(z\right)dz$ (Methods). The resulting *v*_*z*_ profile (Figure S3) shows that fluid within the plumes was moving downward throughout the entire depth of the chamber. Starting from the uppermost surface and moving downward, the flow speed first increased (with the in‐plane flows moving radially inward to the plume center), reaching a peak at a depth of ∼125 μm (∼3/4 of the total height of chamber); then the flow speed decreased gradually to zero all the way toward the bottom, with the in‐plane flows moving radially outward from the plume center. The vertical flow speed profile shows that most flow acceleration occurred at the upper part of the plumes. This result manifests the fact that the driving force triggering the overturning instability (which gives rise to plume formation) is primarily located near the upper portion of the chamber.

### 2.4 Effect of physical parameters on the active plume lattice pattern

The dimensionless Rayleigh number (*Ra*) is commonly used in thermal convection to characterize the ratio of buoyancy‐driven convection to diffusion and controls bioconvection pattern formation [21]. The Rayleigh number is proportional to *n*_0_ *h* ^3^, where *n*_0_ is the average cell number density and *h* is the chamber fluid thickness. Indeed, cell number density and fluid thickness were found in experiments as the key physical parameters controlling the morphology of bioconvection patterns [15, 22]. Therefore, we sought to examine the effect of the two parameters on the plume lattice pattern.

**FIGURE 4:**
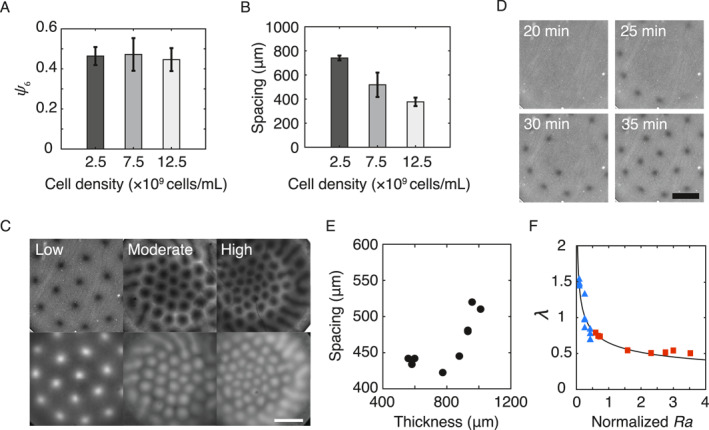
Effect of cell number density and chamber thickness on the active plume lattice pattern. (A) Hexagonal order parameter (*ψ*_6_) of stabilized active plume lattice pattern at different cell densities. (B) Plume spacing at different cell densities. Error bars represent the standard deviation of *N* = 4 biological replicates. (C) Representative phase contrast (upper) and fluorescence (lower) images of the stabilized active plume lattice patterns at low (2.5 × 10^9^ cells/mL), intermediate or moderate (7.5 × 10^9^ cells/mL), and high (12.5 × 10^10^ cells/mL) cell densities. Scale bar, 1 mm. (D) Phase‐contrast microscopy image sequence showing the direct formation of an ordered plume lattice pattern at low cell density. *T* = 0 min corresponds to the starting point of observation when the cell suspension was filled into the chamber and became homogeneous. Scale bar, 1 mm. Also see Video S2. (E) Dependence of plume spacing in the lattice pattern on fluid chamber thickness. (F) The normalized plume wavelength (*λ* = *d*/*h*) as a function of normalized Rayleigh number (*Ra*) in terms of *n*_0_ *h* ^3^ (note that *Ra* ∝ *n*_0_ *h* ^3^). Triangles: *Ra* was tuned by varying cell density *n*_0_ from 2.5 × 10^9^ cells/mL to 1.25 × 10^10^ cells/mL with the chamber fluid thickness fixed at *h* = 500 μm. Squares: *Ra* was tuned by varying chamber fluid thickness *h* from ∼600 μm to ∼1000 μm with cell density fixed at *n*_0_ = 1.25 × 10^10^ cells/mL. Solid line: power‐law fitting in the form of $\lambda \propto {\left(n_{0}h^{3}\right)}^{-\beta }$, with *β* = 0.33 ≈ 1/3 and *R* ^2^ = 0.94.

First, we varied the density of *B. subtilis* cells in the PDMS chamber and found that the plume lattice pattern emerged robustly at cell densities over two orders of magnitude (from ∼5 × 10^8^ cells/mL up to ∼5 × 10^10^ cells/mL). The absence of plume formation at cell densities beyond ∼5 × 10^10^ cells/mL may be due to the suppression of chemotaxis by the collective motion of bacteria [23]. Here, we presented data for stabilized plume lattice in three distinct density regimes (Figure 4): low density (2.5 × 10^9^ cells/mL; Figure 4C, left), intermediate density (7.5 × 10^9^ cells/mL; Figure 4C, middle), and high density (1.25 × 10^10^ cells/mL; Figure 4C, right). The results showed that the order of stabilized active plume lattice pattern did not vary significantly with cell density (Figure 4A). Meanwhile, the mean spacing between plumes decreased with increasing cell density (Figure 4B); this result is consistent with previous studies [15, 22]. Nonetheless, the dynamics of pattern development at low cell densities was different from that shown in Figure 2: The stable plume lattice pattern emerged straight from a homogeneous suspension, without migration and merging of randomly distributed plumes (Figure 4D; Video S2). Next, we varied the thickness of PDMS chambers and found that, while the order of the stabilized active plume lattice pattern was again robust to fluid thickness change, the mean spacing between plumes increased with fluid thickness (Figure 4E), which is consistent with the thickness‐dependence of bioconvection pattern wavelength in green algae *Chlamydomonas nivalis* [22] but at odds with that in an earlier study with *B. subtilis* [15] (see Discussion).

We further examined the relationship between Rayleigh number and the spacing of plumes in the lattice patterns. We compared *Ra* with the normalized plume wavelength *λ* ≡ *d*/*h*, where *d* is the absolute plume spacing and *h* is the chamber thickness [24]. Although there is no analytical solution describing the exact relationship between *Ra* and *λ*, it was found that *Ra* is negatively correlated with *λ* in both experiments and numerical simulations [24]. As shown in Figure 4F, tuning *Ra* by varying either cell density *n*_0_ or chamber thickness *h*, the relation between *λ* and *Ra* falls on the same curve. Interestingly, we found that *λ* approximately scales with *Ra* as *λ* ∝ (*Ra*)^−1/3^.

## 3 DISCUSSION

In this study, we discovered a self‐organized active plume lattice pattern formed by suspensions of chemotactic *B. subtilis* confined in closed 3D fluid chambers. We characterized the pattern developmental process and measured the flow field structure associated with individual plumes. Although the pattern wavelength (i.e., plume spacing) varied with physical parameters including cell density and chamber fluid thickness, the order of the lattice pattern was robust to the variation of these parameters. Remarkably, we uncovered a power‐law scaling relation between the pattern wavelength and the dimensionless Rayleigh number.

Our findings provide experimental evidence that coupling between chemotaxis and gravitational potential field can promote the self‐assembly of regular spatial structures in bacterial populations. The hexagonal active plume lattice pattern observed here was not reported in previous studies of bacterial bioconvection [14, 15, 25, 26]. Previous studies used open fluid chambers, where evaporation‐induced flows might occur and disrupt pattern formation. By contrast, the closed‐chamber geometry we used here eliminates the influence of evaporation‐induced flows and may enable the development of hexagonal order in the distribution of active plumes. The different boundary condition in our experiments may also underlie the difference in thickness‐dependence of bioconvection pattern wavelength from a previous study [15].

A notable feature of the active plume lattice pattern observed here is that the dynamics of pattern development critically depends on cell number density. The pattern development at high cell number densities involves migration and merging of randomly distributed plumes, which is absent for low cell densities (see Figure 4D). We suggest that this result can be understood in terms of the relative strength between hydrodynamics and rotational noise in cell–cell interactions. During cell–cell interactions, it was found that hydrodynamics dominated over noise below a distance called hydrodynamic horizon (*r*_*H*_), which is roughly one cell length (∼5 μm for *B. subtilis*) [27]. This distance corresponds to an average cell number density for *B. subtilis* $n_{0}=1/V_{0}∼1/r_{H}^{3}∼10^{10}$ cells/mL, where *V*_0_ represents the average volume occupied by a single cell. As the cell density goes beyond ∼10^10^ cells/mL, the mean cell–cell distance becomes comparable to or less than *r*_*H*_; in that case, hydrodynamic interaction between cells will be important, potentially inducing collective cellular flows and accounting for the dynamical behavior of plumes at the early stage of plume lattice development.

The ordered plume lattice pattern observed in our study is not only relevant to population dynamics of the species generating the bioconvection pattern but also to the lifestyle of other microorganisms dwelling in the same surface water environments [12, 13]. For instance, the organized vortex structures in the toroidal flows associated with the plumes could affect the dispersal and spatial distribution of microorganisms via rheotaxis [28, 29] or mechanosensing of shear stress [30]. Moreover, the ordered plume lattice pattern as well as the organized flows may be exploited to control self‐assembly in active matter fluids consisting of natural or synthetic microswimmers with taxis behavior.

During preparation of this manuscript, we learned that a recent numerical study on bacterial bioconvection [24] predicted various types of ordered active plume lattice depending on the Rayleigh number, including those similar to the active regular plume lattice we report here; the study also found vortex rings around individual plumes resembling the toroidal convection flow pattern described here. In addition, the simulations found that *Ra* is negatively correlated with *λ*, but the power‐law scaling relation *λ* ∝ (*Ra*)^−1/3^ uncovered in our experiment (Figure 4F) appears to be missing in the model. In addition, the dynamical process of plume development (migration and merging of plumes) was not reported. We noted that the numerical model imposed a slip boundary condition at the top surface, which is different from the no‐slip boundary condition in our experiment. Whether our experimental results can be fully understood in the framework of existing numerical models [24, 26] or require further model development merits further study.

## 4 MATERIALS AND METHODS

### 4.1 Bacterial strains

The following strains were used: *B. subtilis* DS91 (*sinI::spec* [19]); *B. subtilis* DK7921 (*sinI::kan amyE::*P*hag‐gfpmut3 spec*; a *sinI*‐deleted derivative of *B. subtilis* OMG991, which is a gift from Harald Putzer [31]) [32]; an immotile mutant *B. subtilis* DS1677 (Δ*hag*) [33]; *B. subtilis* DK2178 (Δ*cheB amyE::*P*hag‐hagT209C*) [34]; a *sinI* mutant (*sinI::kan*) in *B. subtilis* DK2178 background; and a *sinI*::*kan mutant* in *B. subtilis* DS1122 (*srfAC*::Tn10) background [35]. The latter four strains were used to demonstrate that the bioconvection pattern reported here requires flagellar motility and chemotactic response, but does not require biosurfactant production. Single‐colony isolates were grown overnight (∼14–16 h) in flasks with gyration at 180 rpm in LB medium (1% Bacto tryptone, 0.5% yeast extract, and 0.5% NaCl) at 30°C to the stationary phase.

### 4.2 Fabrication of the microfluidic chamber and preparation of bacterial suspensions

The microfluidic chamber was made of PDMS. To fabricate the microfluidic chamber, a master mold with cylindrical geometry (∼4 mm in diameter and ∼500 μm in thickness) was fabricated on a polymethyl methacrylate plate using the computer numerical control machining technique. Then the master molds were coated with a layer of degassed mixture of silicone elastomer base and a curing agent in a ratio of 10:1 (Sylgard 184, Dow Corning), followed by another 1–2 h degassing with a vacuum pump. The PDMS layer as prepared was cured at 50°C overnight and then cut and separated from the mold. The PDMS was cleaned with transparent adhesive tape (Magic Tape, Scotch). The glass was cleaned with isopropanol by ultrasonic treatment at room temperature for 5 min and then was rinsed with deionized water. The PDMS chamber was then bonded to a glass slide. Meanwhile, PDMS pads were made following the same procedures described above, except that the mixture of the silicone elastomer base and curing agent was poured into 90‐mm polystyrene Petri plates. To prepare the bacterial plume lattice, overnight bacterial cultures were washed by centrifugation (4000 g, 3 min) and resuspended to desired cell number densities. The cell suspension was then pipetted to the PDMS chambers at an appropriate volume (∼10 μL) to fill up the entire volume and then the PDMS chamber was covered with a PDMS pad. For fluid flow measurement, a suspension of fluorescent microspheres (FluoSphere Polystyrene Microspheres, 1.0 μm, red fluorescent (580/605); Invitrogen, Cat. No. F13083) was mixed with the cell suspension to obtain a final concentration of ∼1 × 10^6^ particles/mL.

### 4.3 Microscopy imaging

Imaging was performed on motorized inverted microscopes (Nikon TI‐E or Nikon TI2‐E). The following objectives were used in different experiments for phase contrast and fluorescence imaging: Nikon CFI Plan Fluor DL 4×, N.A. 0.13, W.D. 16.4 mm; Nikon CFI Achromat DL 10×, N.A. 0.25, W.D. 7.0 mm. Fluorescence imaging was performed in epifluorescence with the excitation light provided by an LED illuminator (X‐Cite XLED1; Excelitas Technologies) or a solid‐state light illuminator (SPECTRA Light Engine; Lumencor) using filter sets as follows: a mCherry filter set (excitation 565/55 nm, emission 645/75 nm, dichroic: 600 nm; 49055‐ET‐Wide mCherry/Texas Red for 540–580 nm LEDs; Chroma) for red fluorescent microspheres and an enhanced green fluorescent protein filter set (excitation 470/40 nm, emission 525/50 nm, dichroic: 495 nm; 49002‐ET‐EGFP (FITC/Cy2); Chroma) for fluorescent *B. subtilis* DK7921. Recordings were made with an sCMOS camera [(pco.panda 4.2; PCO) or (Prime BSI Express; Teledyne Photometrics)] using the software NIS‐Elements AR (Nikon). In all experiments, the sample temperature was maintained at room temperature (∼25°C).

### 4.4 Measurement of fluid flow fields

To measure fluid flows, we chose an area of interest with bacterial plume lattice and imaged this area using the 10× objective. To distinguish the velocity fields computed within or outside plumes, fluorescent and phase contrast images were recorded alternatingly: The camera was configured to record at 20 fps and the exposure time for each frame was set as 40 ms; fluorescent and phase contrast images were recorded in alternate frames at 10 fps. Excitation was provided by the LED illuminator (X‐Cite XLED1) through an mCherry filter set (excitation 565/55 nm, emission 645/75 nm, dichroic: 600 nm; 49055‐ET‐Wide mCherry/Texas Red for 540–580 nm LEDs; Chroma). Meanwhile, the bacterial plume lattice was imaged in phase contrast through the same optical system, with the illumination light provided by a white light LED (Cat. No. MCWHL5; Thorlabs). The camera was controlled by NIS elements (Nikon); the white light LED for phase‐contrast microscopy and LED illuminator for fluorescence microscopy were triggered by 10 Hz TTL signals sent from a custom‐programmed Arduino microcontroller that modulated the 20 Hz fire output from the camera. The velocity flow field was obtained by PIV analysis on the image sequences (see section “Image processing and data analysis” below).

### 4.5 Image processing and data analysis

Images processing and data analysis were performed using the open‐source Fiji (ImageJ) software and custom‐written programs in MATLAB (The MathWorks; Natick, Massachusetts, United States).

To calculate the normalized 2D spatial correlation of phase‐contrast images (Figure 2D), we reduced nonuniformity of light illumination and noise by filtering out both low‐ and high‐frequency signals with a custom‐written band‐pass filter program in MATLAB (based on the algorithm of band‐pass filter in ImageJ). The normalized 2D spatial correlation *C*(**r**) is defined as $C\left(r\right)=\frac{\langle \left[I\left(x\right)-\bar{I}\left(x+r\right)\right]\left[I\left(x+r\right)-\bar{I}\right]\rangle }{\sqrt{\langle {\left[I\left(x\right)-\bar{I}\left(x+r\right)\right]}^{2}\rangle \langle {\left[I\left(x+r\right)-\bar{I}\right]}^{2}\rangle }}$ over the image [36], where *I*(**x**) denotes the grayscale value of the phase‐contrast image at position **x**, $\bar{I}\left(x+r\right)$ denotes the mean grayscale value in a window of the same size as the image centered at position **x** + **r** in the image, $\bar{I}$ denotes the overall mean grayscale value of the image and the angular bracket represents the ensemble average over the image. It is worth noting that when calculating the mean grayscale value $\bar{I}\left(x+r\right)$ for windows extending beyond the image boundaries, the values outside the image were treated as zero. The normalized 2D spatial autocorrelation was computed using the built‐in function *“normxcorr*2*”* in MATLAB. To obtain the radially averaged spatial correlation function (Figure 2A), the resulting normalized 2D spatial autocorrelation maps were then radially averaged to yield $C\left(r\right)=\left[\int_{r}^{r+∆r}C\left(r\right)dr\right]/(2\pi r∆r)$.

We identified the plume regions in a phase‐contrast image following the steps below: (1) Reduce the nonuniformity of light illumination and noise by filtering out both low‐ and high‐frequency signals with a custom‐written band‐pass filter program in MATLAB (based on the algorithm of band‐pass filter in ImageJ); (2) Binarize the band‐pass‐filtered image using Otsu’s method [37], which automatically determines a global threshold by minimizing the intraclass variance of the black and white pixels. The resulting binary images suggest the plume regions, with plumes represented as white pixels (value of 1) and non‐plume regions as black pixels (value of 0). The centers of the plumes were then obtained as the centroid coordinates of each connected white pixel regions in each binary image for the calculation of hexagonal order parameter.

The hexagonal order parameter of plume lattice pattern is defined as $\psi_{6}=\langle {|\psi }_{6}^{m}|\rangle$, with $\psi_{6}^{m}=\frac{1}{N}\sum_{n=1}^{N}e^{6i\theta_{nm}}$ [38]; here *N* is the number of nearest neighbors of plume *m*, *θ*_*nm*_ is the angle between a reference horizontal axis direction and the direction pointing from the center of plume *m* to the center of its neighboring plume *n*, and the angular bracket represents ensemble average over plume index *m* in the entire plume lattice pattern.

To compute the flow field traced by microspheres at different heights, PIV analysis was applied to fluorescence microscopy images using a custom written program in MATLAB based on PIVlab [39]. For a faster computation, we chose each pair of consecutive images of time interval 0.5 s. For each pair of consecutive images obtained with the 10× objective, the PIV interrogation‐window size started at 41.6 μm × 41.6 μm and ended at 20.8 μm × 20.8 μm after two iterations. The resulting velocity field **v**_plane_(**r**, *t*)=(*v*_*x*_, *v*_*y*_) was then smoothed by first removing the obtained velocity vectors with a magnitude greater than the local mean magnitude by five times of the standard deviation and then interpolating the missing vectors in the field. The in‐plane divergence of velocity flow field (defined as ∇⋅**v**_plane_ = ∂_*x*_ *v*_*x*_ + ∂_*y*_ *v*_*y*_) was computed by the function “*divergence”* in MATLAB. To compute the divergence probability distribution within plumes, we first manually labeled the plume regions and then counted the pixelwise divergence within these labeled plume regions. To obtain the actual height *H* of the focal plane, we first focused on microspheres located at the bottom of the sample and recorded the position of the objective lens *z* as a reference plane, with the height at this point set as *z* = 0. Then we tuned the objective lens to focus on the desired focal plane and recorded the displacement Δ*z* from the reference plane. The actual height *H* of the focal plane equals the vertical displacement Δ*z* multiplied by the refractive index of water (∼1.33) as *H* = 1.33Δ*z*.

To compute the vertical velocity (*v*_*z*_) as a function of height (Figure S3), the mean divergence was interpolated by spline interpolation between two consecutive mean divergence values presented in Figure 3B. The incompressibility of the fluid dictates that the total divergence must equal zero, that is, ∇⋅**v** = 0. This total divergence is the sum of the in‐plane divergence (*D*_in_) and the gradient of *v*_*z*_ in vertical direction (∂_*z*_ *v*_*z*_, with +*z*‐axis pointing vertically upward), as expressed by the equation ∇⋅**v** = ∂_*x*_ *v*_*x*_ + ∂_*y*_ *v*_*y*_ + ∂_*z*_ *v*_*z*_ = *D*_in_ + ∂_*z*_ *v*_*z*_ = 0. By integrating the interpolated mean in‐plane divergence over the heights (Figure S3A), we can estimate *v*_*z*_ at a different height *H* above the bottom of the chamber by $v_{z}\left(H\right)-v_{z}\left(0\right)=-\int_{0}^{H}D_{\text{in}}\left(z\right)dz$. To ensure that *v*_*z*_(*H*) is negative near *H* = 0 (i.e., fluid near the bottom of the plumes moving downward due to entrainment by downward moving cells), we need to set *v*_*z*_(*H* = 0) = 0 (note that *v*_*z*_ cannot be negative at *H* = 0).

## AUTHOR CONTRIBUTIONS

**Siyu Liu**: Conceptualization; data curation; formal analysis; investigation; methodology; writing—original draft. **Qihui Hou**: Methodology. **Daniel B. Kearns**: Funding acquisition; methodology; resources. **Yilin Wu**: Conceptualization; formal analysis; funding acquisition; investigation; resources; supervision; writing—review & editing.

## CONFLICT OF INTEREST STATEMENT

The authors Siyu Liu, Qihui Hou, Daniel B. Kearns, and Yilin Wu declare that they have no conflicts of interest or financial conflicts to disclose.

## ETHICS STATEMENT

This article does not contain any studies with human or animal materials performed by any of the authors.

## Supporting information

Supporting Information S1

Video S1

Video S2

## Data Availability

The authors declare that the data supporting the findings of this study are available within the paper and its supplementary information files.
