# Supplementary material for: Self‐organization of active plume lattice in bacterial bioconvection
Source: Quant Biol. 2024 Dec 18;13(1):e80. doi: 10.1002/qub2.80 (PMC12806025; doi:10.1002/qub2.80)
Supplement: Supplementary file 1 — Supporting Information S1 [file QUB2-13-e80-s001.pdf]

Supplementary Information (SI) for

Self-assembly of bacterial suspensions into plume lattices via bioconvection

Siyu Liu [1], Qihui Hou [1], Daniel B. Kearns[2], Yilin Wu [1*]

[1] *Department of Physics and Shenzhen Research Institute, The Chinese University of Hong Kong, Shatin, NT, Hong Kong, P.R. China.*

[2] *Department of Biology, Indiana University, Bloomington IN, USA*

The SI includes the following sections:

1. SI Figures (Fig. S1-S3)

2. Legends for Supplementary Video S1-S2

SI figures

Figure S1

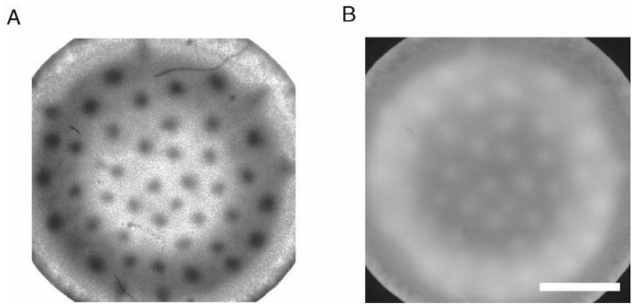

Fig. S1. Active plume lattice pattern of a *B. subtilis* suspension confined in a closed 3D fluid chamber. (A) A representative phase-contrast microscopy image. (B) Fluorescence microscopy image of the same field of view as in panel A. Since all cells were labeled by fluorescent protein, this result shows that the darker domains have a higher cell number density than their surroundings. Scale bar, 1 mm.

Figure S2

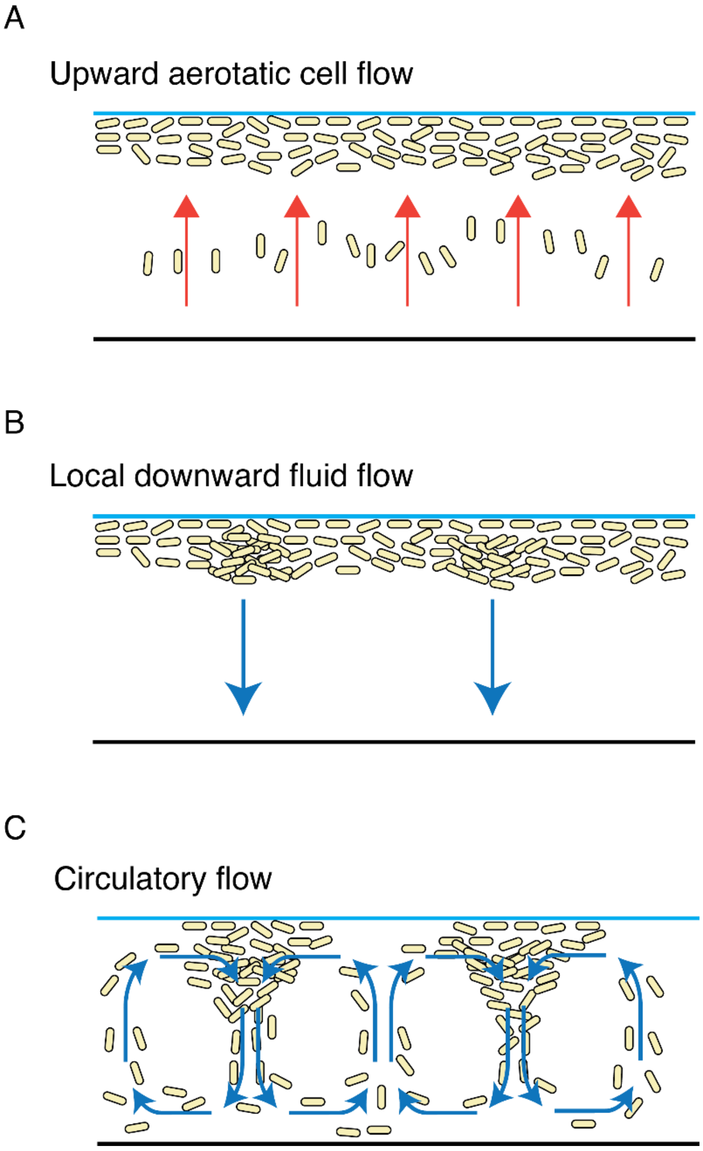

Fig. S2. Schematic illustration of the bioconvection pattern formation process. (A) Cells migrate upwards due to taxis behavior, accumulating at the upper surface. (B) Fluctuations lead to localized increase in cell number density at the upper surface, where the denser aggregates of cells experience a downward negative buoyancy force due to the higher mass density than the surrounding fluid. (C) Vertical columnar structures (or active bacterial plumes) develop, with cells falling downward and being pumped outward near the base, recirculating upward at the periphery, and finally moving inward towards the center of columnar structures near the top surface.

Figure S3

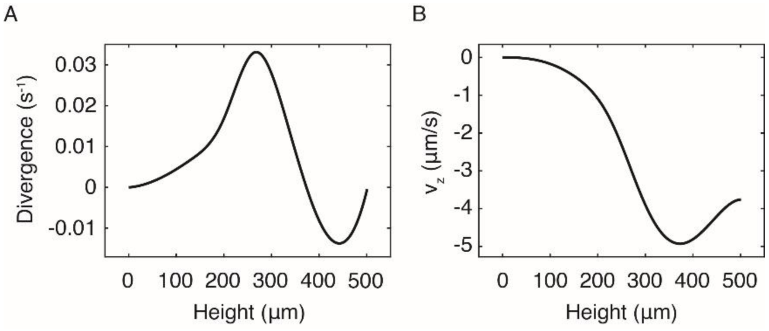

Fig. S3. Mean in-plane divergence and vertical velocity in plumes as a function of chamber height. (A) Mean in-plane divergence D_{in} within plumes as a function of height above the bottom of the chamber. The mean in-plane divergence was computed by spline interpolation between two consecutive mean in-plane divergence values presented in Fig. 3B. (B) Vertical velocity profile as a function of height above the bottom of the chamber. The vertical velocity v_z at different heights H was computed by integrating the interpolated mean divergence $D_{\mathrm{in}}(H)$ from panel A as $v_z(H) = -\int_0^H D_{\mathrm{in}}(z)dz$ (Methods).

Legends of supplementary videos

Video S1. Dynamics of active plume lattice development. This phase-contrast video is associated with Fig. 1 in main text. The time elapsed in the experiment is indicated by the time stamp (format: hh:mm:ss). Scale bar, 500 μm.

Video S2. Developmental process of ordered bacterial plume lattice at low cell density (2.5×10^9 cells/ml). Time-lapse imaging by phase-contrast (left) and fluorescence (right) microscopy captures the spontaneous formation of the plume lattice without migration and merging of plumes. This video is associated with Fig. 4D in main text. Scale bar, 1 mm.
